# Assessment of Landing Parameters in Patients with Chronic Ankle Instability

**DOI:** 10.3390/jcm14176074

**Published:** 2025-08-28

**Authors:** Małgorzata Kowalczyk, Aleksandra Truszczyńska-Baszak, Krzysztof Dudziński, Barbara Łysoń-Ukłańska

**Affiliations:** 1 Doctoral School, Józef Piłsudski University of Physical Education in Warsaw, 00-968 Warsaw, Poland; kontakt@efizjo.pl; 2 Faculty of Rehabilitation, Józef Piłsudski University of Physical Education in Warsaw, 00-968 Warsaw, Poland; krzysztof.dudzinski@awf.edu.pl (K.D.);

**Keywords:** chronic ankle instability, Coper, time to stability, proprioception, balance, neuromuscular control

## Abstract

**Background:** The aim of this study was a qualitative assessment and quantitative assessment, i.e., the assessment of time to stability (TTS) before and after fatigue test, of landing in patients with chronic ankle instability, referred to as “copers”, in comparison to a control group of healthy persons. The indirect aim of the study was to develop a new method to assess more time precise measurement of TTS. **Methods:** The study involved 60 physically young active individuals aged 18 to 35 years. They were divided into three groups: the study population of 29 copers was divided into: I—14 persons with chronic one side ankle instability, study population II—15 persons with chronic bilateral ankle instability, and the control group included 31 persons without ankle instability. The study involved quantitative assessment of time to stability (TTS) after single-leg landing onto the HUR stabilometric platform from a 30 cm high platform before and after fatigue tests based on a modified Short-Term Fatigue Protocol. To conduct qualitative assessment of landing and to verify time to stability with video imaging, a video analysis was conducted. We used three cameras and two markers: on the malleolus lateralis of the fibula and on the malleolus medialis of the tibia. Each landing was subjectively assessed by a physiotherapist on a four-degree scale. A further biomechanic analysis was conducted only for the trials with a correct landing. The trials were repeated after a fatigue test. **Results:** There were significant differences before and after the fatigue test in qualitative landing analysis (*p* < 0.001) only for one jump for the right leg. In groups with unilateral and bilateral ankle instability, there was a higher percentage of landings with a considerable shift or unstable landings. The conducted dynamic test (TTS assessment) did not reveal significant differences between groups or after the fatigue test. **Conclusions:** Copers develop effective mechanisms of compensation, allowing them to participate in physical activities without symptoms of joint instability. Determining biomechanical differences between athletes who return to their sport and patients who develop chronic instability is important in the context of introducing adequate physiotherapy.

## 1. Introduction

One of the most common absence reasons in sport activities are inversion injuries of the foot (25%) [1,2,3]. Incorrect treatment or lack of treatment result in recurring injuries [4], and a consequence may be a development of chronic ankle instability (CAI) [5,6,7,8]. CAI is characterized by repeated episodes of ankle instability that can develop as soon as after the first inversion injury [9]. Interestingly, there is a group of persons who, despite experiencing earlier inversion injury or injuries, do not develop symptoms that would disqualify them from doing sports on a high level. Such sportspeople are referred to as “copers” [10]. It seems that they can develop efficient compensatory mechanisms that protect them from having subsequent injuries. One of the factors for repeated inversion injuries is muscle fatigue. Fatigue may lead to incorrect positioning of the lower limb and in difficulties in fast reaction to changes in direction of movements, therefore leading to injuries [10,11,12]. Injury risk for subjects with CAI may be significantly higher [13].

One of the studies conducted on sportspeople is an assessment of various parameters in landing from a height onto the dysfunctional extremity. In this case, the landing imitates a situation in which numerous inversion injuries occur. These parameters include inter alia an assessment of angles in lower and upper ankle joints, knee joints, and hip joints in landing [14,15,16], as well as a qualitative assessment of landing and time to stability assessment after landing [17].

The aim of this research was a qualitative and quantitative assessment, i.e., the assessment of time to stability (TTS) before and after a fatigue test, of landing in patients with chronic ankle instability, referred to as “copers”, in comparison to control group of healthy persons. The indirect aim of the study was to develop a new method to assess TTS.

## 2. Material and Method

All recommendations outlined in the STROBE statement were followed. Study protocols were approved by the Józef Piłsudski University of Physical Education in Warsaw Senate Research Ethics Committee no. 01-19/2017, date 5 May 2017. All investigations were conducted in accordance with the principles outlined in the Declaration of Helsinki. All participants signed informed consent for participation. The research protocol was conducted at the Centre for Functional Diagnostics at Carolina Medical Center in Warsaw, Poland between 2018–2020.

The study involved 60 physically active individuals aged 18 to 35 years. They were divided into three groups (Table 1 and Table 2 present group characteristics):

- Study population I—14 individuals with instability of one ankle,
- Study population II—15 individuals with instability of both ankles,
- Control group—31 individuals, without joint instability.

The G*Power 3.1.9.4. program was used to check if the test power of results was sufficient. The effect size was calculated based on the general stability index for the three groups (f = 0.47). We used an analysis of variance scheme in the mixed pattern 3 × 2 (3 groups × 2 measurements) with α = 0.05 to conduct the calculations. The estimated test power was 0.94, which proves that the studied sample was sufficient to conduct the analyses. 

Criteria for subject inclusion in the study population:

- Young adults (below 35 years old);
- Minimum of 2 ankle torsions to the same extremity within the past 3 years (with a minimum of one serious torsion causing impossibility to sport participation ≥ 21 days);
- Tear of anterior talofibular ligament and/or talocalcaneal ligament III°, confirmed by ultrasound and/or MRI;
- Minimum 60 points on The Foot & Ankle Disability Index Score (FADI) and on the sports module (FADI-S);
- Return to sport on previous level for over 6 months;
- Participation in an endurance sport a minimum of twice a week.

Criteria for subject inclusion in the control group:

- Young adults (below 35 years old);
- No ankle injuries in history;
- Participation in an endurance sport a minimum of twice a week.

Exclusion criteria for all groups: All cardiological, orthopedic and neurological contraindications for performing fatigue test and for training sports professionally.

All participant fulfilled FADI and FADI-S questionaries. Initially, 74 subjects were included in the study. Eight subjects did not show up for the tests, and six did not finish all tests.

The tests were performed at the Centre for Functional Diagnostics of Carolina Medical Centre in Warsaw. The person conducting the tests (M.K.) is a physiotherapist with 10+ years of experience.

### 2.1. Study Protocol

The warm-up consisted of 10 min cycling on a stationary bike as well as a 5 min-long exercise of the subjects’ choice. Then, the maximum jump height was determined (Hmax). The subjects performed three vertical countermovement jumps (CMJ) on the AMTI dynamometer platform. The highest jump value from three trials (Hmax [in cm]) was chosen for analysis.

Then, the subject performed three single-leg landings onto the HUR stabilometric platform from a 30 cm high platform. The test was combined with a video analysis incorporating 3 high-speed cameras (100 Hz) and Simi Motion software 8.5.338. We used two markers placed on the following anatomical points: on the malleolus lateralis of the fibula and on the malleolus medialis of the tibia to conduct qualitative assessment of landing and to verify time to stability with video imaging.

To compare the control group and study populations I and II according to the qualitative assessment of landing, we used Fisher’s exact test. We conducted separate analyses for assessments for right unstable leg landing in the study populations I and II and for the stable leg in the control group; and for the left unstable lower extremity in the study populations I and II and for the stable in the control group.

Each landing was subjectively assessed by a physiotherapist based on the registered video material and awarded an adequate score:

1. Correct landing;
2. Landing with a slight shift during landing (up to 1/2 foot width);
3. Landing with a significant shift during landing (>1 foot width);
4. Unstable landing.

A further biomechanic analysis was conducted only for the trials with correct landing.

### 2.2. Time to Stability—Quantitative Assessment

This parameter is a quantitative measurement of force used for measuring dynamic postural stability [17]. The TTS measure indicates how quickly a person regains stability after jumping and it is used for quantitative determination of postural stability after jumping. The study aimed to design a new method for TTS assessment that could allow for a more time precise measurement of TTS.

**Figure 1:**
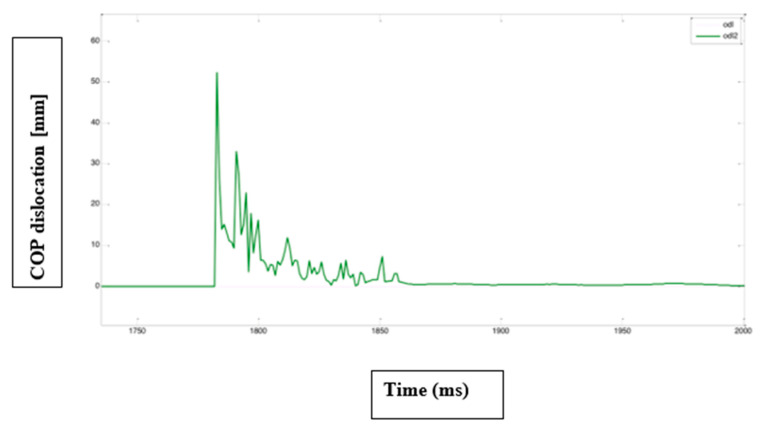
Time to stability was determined based on path of center of foot pressure COP.

**Figure 2:**
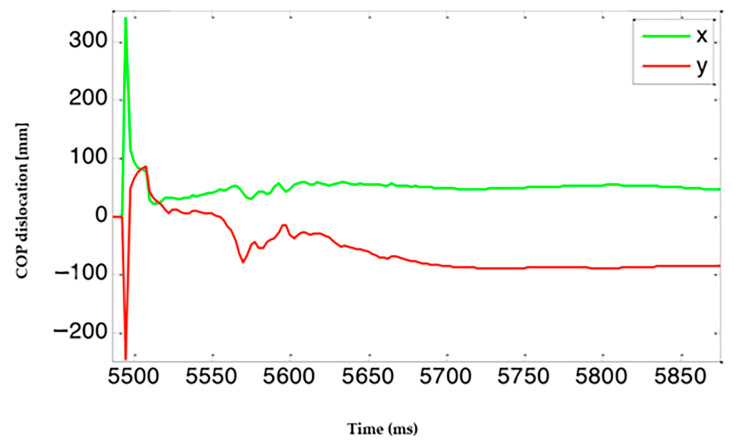
Determining global COP shift.

**Figure 3:**
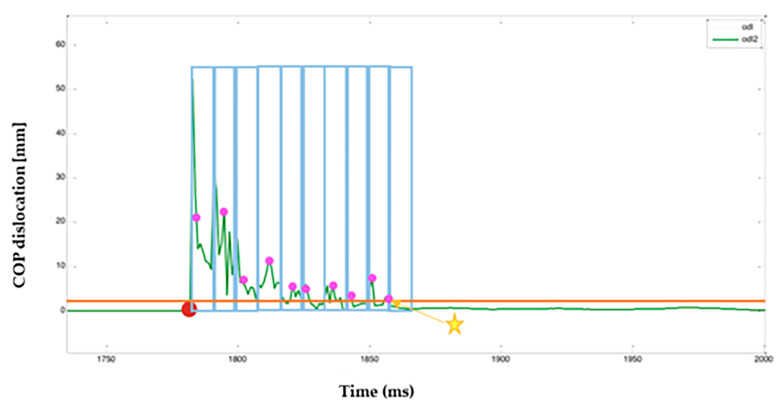
Determination of contact point of extremity with platform (a sudden increase of COP shift—red dot on of Figure 3). Red dot—the moment of limb contact with the measurement platform was identified, characterized by a sudden increase in COP displacement. Subsequently, the COP displacement signal was divided into time windows of 20 samples each (indicated by blue rectangles). Within each window, the value exceeding 95% of the samples in that window was determined (marked with a pink dot). These values were then compared to a predefined threshold (orange line). The beginning of the first window for which the value fell below the threshold was identified as the stabilization time (marked with a yellow star).

Our study used a dynamometric platform. Time to stability was determined based on the path of center of foot pressure COP [mm] in time (Figure 1). The test provided two COP paths in time for the X axis and Y axis, which were processed by a medical bioengineer in the MATLAB R2018b software in the following procedure:

(1) Determining the global COP shift (Figure 2);
(2) Based on the obtained path, determination of the contact point of the extremity with the platform (a sudden increase of COP shift—red dot on of Figure 3);
(3) Establishing a divide of the path into time windows (window size—20 samples) with blue rectangles on and within each window, determining a value that was greater than 95% of samples within a window;
(4) A comparison of the value obtained in the previous step with the threshold (orange line on Figure 3); the beginning of a window for which the value was lower than the threshold was determined to be the time to stability.

During the analysis of COP signal, we tested numerous thresholds and decided on the sample which was adequate for the visual and subjective assessment of the signal going silent. This was additionally verified against the video analysis. The method was chosen so that the isolated placement of the COP path below the threshold did not result in determination of stability. The condition for stability of the landing was that the COP shift within window was below threshold for 95% of window length.

The COP shift was determined based on the following formula:

(1) $$d=\sqrt{{\left(x_{i}-x_{i-1}\right)}^{2}+{\left(y_{i}-y_{i-1}\right)}^{2}}$$

where *x_i_*, *x_i_*_−1_ are coordinate x for COP signal for the present and following sample, and *y_i_*, *y_i_*_−1_ are coordinate y for COP signal for the present and following sample (Figure 3).

After the landing analysis, the subjects performed a fatigue test based on the modified Short-Term Fatigue Protocol (FAST-FP) [18]. The fatigue test was described in an article by Kowalczyk and Truszczyńska-Baszak (2023) [19].

Criteria for ending the fatigue tests were not reaching a minimum of 90% of max height in one of three jumps, over 90% of HRmax = 202.5–0.53 × subject’s age, or refusal to continue the trial.

After the fatigue test, another landing analysis was performed for each study participant.

## 3. Results

### 3.1. Qualitative Assessment of Landing Before and After Fatigue Test

The conducted analysis revealed statistically significant difference between the groups only for jump 1 for right leg. Among individuals from the control group, there were significantly more correct landings than among individuals from study populations I and II. The percentage of correct landing on stable right leg was higher than on the unstable right leg in study populations I and II. In study population I, the percentage of individuals whose landing was assessed as having a significant shift was significantly higher than in the control group. In study population II, the percentage of unstable landings was significantly higher than in the control group (Table 3); in study population II it was the right unstable leg, while in the control group, the stable right leg. For the remaining landings, the differences in qualitative assessments between groups proved to be insignificant (Table 3, Table 4, Table 5 and Table 6).

There is a difference on the level of *p* < 0.05 between the columns which do not share the letter index.

### 3.2. Time to Stability—Quantitative Assessment Before and After Fatigue Test

To verify the differences between groups regarding the mean from three jumps in study populations I and II and in the control group for the right and left leg, we conducted variance analysis in the mixed scheme 3 × 2. The analysis excluded unstable jumps and jumps with a significant shift, i.e., shift greater than foot width.

The first analysis included a comparison of means right leg. This leg was unstable in study group I and II, and stable in the control group. The compared groups were homogenous in terms of analysis of variance.

The conducted analysis did not reveal main effect for the mean of three jumps for right leg, F(1.43) = 0.08; *p* = 0.784; η_p_^2^ = 0.01. This means that the mean for three jumps before (M = 1.47; SE = 0.07) and after fatigue test (M = 1.50; SE = 0.10) were statistically insignificant.

The main effect for the group also proved not to be significant, F(2.43) = 0.54; *p* = 0.587; η_p_^2^ = 0.02. Group I (M = 1.40; SE = 0.18), group II (M = 1.58; SE = 0.10) and the control group (M = 1.47; SE = 0.06) did not differ regarding the mean from three jumps for the right unstable leg.

The interaction of both variables, the group and the measurement, proved insignificant, too F(2.43) = 1.41; *p* = 0.255; η_p_^2^ = 0.06. We did not find differences between measurements before and after fatigue test within group I (F(1.43) = 0.28; *p* = 0.600; η_p_^2^ < 0.01), group II (F(1.43) = 1.29; *p* = 0.262; η_p_^2^ = 0.03), and the control group (F(1.43) = 1.61; *p* = 0.212; η_p_^2^ < 0.01).

The differences between groups measurements taken before fatigue test (F(2.43) = 2.51; *p* = 0.093; η_p_^2^ = 0.10) and after fatigue test were insignificant (F(2.43) = 0.03; *p* = 0.968; η_p_^2^ = 0.01). This means that for both measurements the differences between groups regarding mean jump length for the unstable leg (groups I and II) and the control group proved insignificant. Results are shown in Table 7.

Identical analyses were conducted for the left leg. Left leg was unstable in groups I and II, and stable in the control group.

The conducted analysis did not reveal main effect for the mean of three jumps for left leg, F(1.45) = 0.12; *p* = 0.729; η_p_^2^ = 0.01. This means that the mean for three jumps before (M = 1.47; SE = 0.08) and after fatigue test (M = 1.44; SE = 0.08) were statistically insignificant.

The main effect for the group also proved not to be significant, F(2.45) = 0.83; *p* = 0.444; η_p_^2^ = 0.04. Group I (M = 1.33; SE = 0.15), group II (M = 1.56; SE = 0.10) and the control group (M = 1.46; SE = 0.07) did not differ regarding the mean from three jumps for the left unstable leg.

The interaction of both variables, the group and the measurement, proved insignificant, as well (F(2.45) = 0.23; *p* = 0.729; η_p_^2^ = 0.01). We did not find differences between the measurements of group I (F(1.45) = 0.04; *p* = 0.817; η_p_^2^ < 0.01), group II (F(1.45) = 0.62; *p* = 0.434; <0.01), and the control group (F(1.45) = 0.08; *p* = 0.775; η_p_^2^ < 0.01). 

The differences between groups measure taken before test (F(2.45) = 1.14; *p* = 0.330; η_p_^2^ = 0.05) and after proved insignificant (F(2.45) = 0.19; *p* = 0.830; η_p_^2^ = 0.01). This means that for both measurements the differences between groups regarding mean jump length for the unstable leg (I and II) and stable leg (control group) proved insignificant. Descriptive statistics are presented in Table 8.

## 4. Discussion

The aim of the study was the qualitative assessment of landing as well as quantitative assessment of time to stability (TTS) after single leg landing with our proprietary method and assessment of the usefulness of this method in differentiating between healthy individuals and individuals after inversion injury who returned to sports activity from before the injury, so called “copers”, under the influence of fatigue in studied groups. In groups with unilateral and bilateral ankle instability, there was a higher percentage of landings with considerable shift or unstable landings. The conducted dynamic test (TTS assessment) did not reveal significant differences between groups or after the fatigue test.

Development of a method that could quantitatively determine dynamic postural stability would help the clinicians to identify individuals with higher risks of inversion injury. It could also provide a screening test that would inform of dynamic postural stability in sports persons before the start of the sports season. One of the tests that determines dynamic postural stability discussed in the literature is the time to stability method (TTS), originally designed by Ross and Guskiewicz [17]. TTS determines the time from the initial ground reaction forces (GRF) to the free stance. The TTS measurement indicates how quickly a person stabilizes after landing from a jump. Although quite recent, the TTS is one of the still few measurements of dynamic postural stability which detects differences between individuals with stable and unstable joints during dynamic tests [18]. Wright et al. [20] determined TTS as time after landing to reaching stability of ground reaction forces (GRF) in the antero-posterior plane or the medio-lateral plane to the level of normal stance.

In the published studies, TTS was measured during landing as it is a dynamic activity, requiring a swift halt of the movement by lower extremities so that the body is stabilized [17]. Additionally, inversion injuries of the ankle usually occur during landing, especially when an individual is fatigued [21]. Moreover, several scientists have speculated that as they present potential reasons for functional instability, i.e., damaged ligaments, muscle strength deficits, lowered muscle reaction time, or deficits in proprioception, individuals with CAI may present different landing patterns after jump in comparison to individuals with stable ankles [20,22,23]. Quick stabilization after landing is necessary in many sport disciplines based on jumping, e.g., volleyball, basketball, or skiing. It is believed that ability to stabilize swiftly after jump may minimize the risk of injury [17].

Doherty et al. [24] proved that time to stability in group if individuals with functional instability was longer (1.98 ± 0.81 s) in comparison with a group of healthy controls (1.45 ± 0.30 s) (*p* < 0.05). We did not find any studies in the available literature that would determine time to stability in the range of ballistic movement, when an injury occurs. This is why we aimed at designing a new method that would assess TTS determined based on center of foot pressure shifts in time, and not, as had been done before, on the ground reaction forces. Even though we used a different parameter to determine time to stability after landing, the time necessary to silence the COP signal was shown to be similar to the times published in the literature.

Some of the studies confirm the significant differences in TTS between individuals with CAI and healthy controls; individuals with CAI need more time to stabilize posture after landing [24,25,26]. Other studies, similarly to our study, do not seem to confirm this tendency [27,28]. The differences may have resulted from differences in study populations. Our study assessed “copers”, individuals who had developed effective compensation mechanisms. Finding biomechanic differences between “copers” and healthy controls with the used of available measurement devices is a challenging task. The differences between these groups seem to be very subtle.

In our method, we tested numerous threshold window sizes during the analysis of COP signal, and we decided on the sample which was adequate for the visual and subjective assessment of the signal going silent. This was additionally verified against the video analysis. The condition for stabilization of the landing after jump was that the COP shift within the window was below threshold for 95% of window length. In contrast, GRF signal analysis by Ross et al. [17] did not correspond to the visual assessment of GFR silencing. The researchers scanned the last 10 s from each trial (10–15 s and 15–20 s). The time window which was characterized with a smaller scope of GRF variability reflected the optimal postural stability of the studied individual. After the time window was chosen, the highest value of GRF amplitude was the horizontal line on the chart of ground reaction forces. Then, the third-degree polynomial was determined. The intersection of the polynomial and the horizontal line determined the time to stability in seconds [s].

The methods for calculating TTS discussed so far had used the range of ground reaction forces GRF variability in single-leg free stance of the studied individual as the reference value for calculating TTS [17,24,28]. Goldie et al. [29] proved that individuals with ankle instability have greater range of ground reaction forces variability in single-leg free stance than individuals with stable joints. Applying the above-mentioned calculations may not reveal differences between individuals with joint instability and healthy controls because the reference value for individuals with CAI is their own stability level [17]. This was the reason why Ross et al. [17] suggested that normalized referencing variables that are not affected by instability should be used for calculating TTS. Therefore, the study by Ross et al. [30] normalized all the results to the levels of normal standing of healthy subjects. The assumptions of these authors seem justified, yet there seemed to have been an issue with the small size of the study population on which the authors normalized the published results, as it was only 10 individuals. A methodology assessment indicates that this is unreliable. Our study determined the stability time to the level of normal stance of studied individuals. This was because, during the experiment, the studied individuals had not been instructed to maintain the position for a certain amount of time e.g., 10–15 s after landing. The assumption of the new method was to assess time to stability in a very short time after landing, within the range of ballistic movement. Unfortunately, result analysis in the studied groups showed that the COP signal does not silence as quickly as it was assumed after analysis of results from the pilot study. It may therefore be believed to be the reason for lack of differences between groups in TTS in our study.

Another difference applied in our study and in the studies by Ross et al. [17] and Wikstrom et al. [31] was the type of landing protocol used during TTS measurements. The studies by Ross et al. and Wikstrom et al. used a forward jump landing protocol onto a platform from the height of 50% of the subject’s maximum height and the distance of 70 cm. In their study, Steib et al. [27] changed the direction of landing, as it used diagonal jump = landing maneuver because it better reflected the dynamic conditions typical for sports activity or injury occurrence. Our study used jump from a 30 cm high platform, which meant that the landing conditions were the same for every participant.

In our study, the calculations and TTS quantitative analysis did not involve jumps which resulted in significant shift or which was unstable. This data was processed within qualitative analysis. Based on the registered video material, each landing was subjectively assessed by a physiotherapist and awarded an adequate description (1. correct landing, 2. slight shift, 3. significant shift, 4. unstable landing). Significant differences between groups were observed. Study population I (unilateral instability) had a considerably higher percentage of individuals whose landing on the unstable extremity had a significant shift that the control group, while in study population II, the percentage of unstable landings was considerably greater than in the control group. An increased percentage of landings with considerable shift and of unstable landings reveals changed movement kinematics during landing and weakened compensation mechanisms, which probably increase injury risk. Numerous studies have found several kinematic changes that increase the probability of injury during landing [32,33,34]. Cortes et al. [35] found changes to the kinematics of lower extremity such as decreased flexion angle of the knee and the hip during landing (a more straightened body position), in their comparison of individuals with CAI with healthy controls. Quammen et al. [18] proved similar biomechanical changes in their study on healthy professional football players. A study by McLean et al. [33] found that in individuals with CAI in the early phase of landing the foot had greater plantarflexion and supination and that these changes were more intense in women. Additionally, a study by Wright et al. [25] reported that patients with CAI during landing presented bigger dorsiflexion ROM in ankle joint. Our study purposefully resigned from kinematic assessment during landing because of the time criterion in the stress test. The main aim of this study was an assessment of individual kinematic changes under fatigue. In order not to allow for regeneration after stress test, it was essential to conduct the trials as quickly as possible after fatigue test. This was why we decided not to perform kinematic analysis; it would not allow for measurements in the state of fatigue. Moreover, kinematic analysis of individuals with CAI has been thoroughly studied and extensively published in the literature [14,15,16].

Risk of injury increases with fatigue that may lead to changes in lower extremity biomechanics during landing [20]. Fatigue leads to decreased muscular maximum voluntary contraction and decreases proprioception [35,36,37].

### Limitation of the Study

In real-life situations during a match or sporting activity, athletes are exposed to constantly changing and demanding conditions requiring unexpected and multitasking movements. The study design did not allow participants to utilize various techniques for jumping, landing, or stabilizing after the jump. A controlled movement task was used during which the participant focused solely on the intended movement and could plan it, which is not possible in natural settings, especially team sports. Landing strategies in laboratory conditions may therefore differ from those used during on-field sporting activity.

Additionally, to better reflect real-world conditions, studies should include an unexpected movement task, which would eliminate the participant’s pre-planning to some extent and demonstrate more changes in neuromuscular control.

This study utilized a fatigue protocol, in which the number of sets was determined by the individual endurance predispositions of the subject. Despite the use of high extrinsic motivation during the exercise test, some subjects may not have exerted maximum effort, which could also have influenced the study results.

## 5. Conclusions

Copers develop efficient compensatory mechanisms that enable them to return to sport without clinical symptoms instability or re-injury. Determining biomechanical differences between individuals who regain high sports activity levels and those who develop CAI instability is important in the context of introducing adequate physiotherapy. Undoubtedly there is a need for further studies involving measurement methods that would to a greater extent reflect the realistic conditions of sports activities, and therefore will allow for identifying differences between these groups of patients.

## Figures and Tables

**Table 1:** Biometric data of studied groups. All differences between groups were statistically insignificant.

Variable	Study Population I (*n* = 14; 5 K, 9 M)	Study Population II (*n* = 15; 5 K, 10 M)	Control Group (*n* = 31; 10 K, 21 M)
Age (years)	29.8 ± 4.6 21.0 ÷ 35.0	30.3 ± 5.0 18.0 ÷ 35.0	29.8 ± 3.4 24.0 ÷ 35.0
Body height (cm)	180.0 ± 9.0 160.0 ÷ 189.0	180.0 ± 10.0 170.0 ÷ 192.0	176.4 ± 9.1 161.0 ÷ 195.0
Body mass (kg)	70.0 ± 11.2 54.0 ÷ 82.0	76.1 ± 13.5 55.0 ÷ 100.0	74.5 ± 13.0 50.0 ÷ 107.0
BMI	22.8 ± 2.0 18.7 ÷ 25.2	22.3 ± 3.6 18.0 ÷ 30.2	23.8 ± 2.9 18.4 ÷ 32.0

**Table 2:** Characteristics of weekly physical activity and FADI/FAD-S of study groups (I, II, III).

Variable	Study Population I (*n* = 14)	Study Population II (*n* = 15)	Control Group (*n* = 31)
Weekly physical activity (hours)	4.1 ± 1.9 2.0 ÷ 8.0	4.8 ± 3.2 2.0 ÷ 12.0	5.3 ± 2.6 2.0 ÷ 15.0
FADI	96.4 ± 3.6 90.4 ÷ 100.0	97.1 ± 4.0 88.5 ÷ 100.0	99.4 ± 2.4 86.5 ÷ 100.0
FADI-S	90.6 ± 8.5 * 75.0 ÷ 100.0	91.4 ± 10.4 * 71.9 ÷ 100.0	98.7 ± 5.1 * 71.9 ÷ 100.0

* Significant differences (*p* < 0.05) between group I, II and the control group.

**Table 3:** Analysis of frequency with Fisher’s exact test for qualitative assessment of landing before fatigue test—right lower extremity (*p*—test probability of Fisher’s exact test; *V*—Cramer’s—effect size, number in brackets gives % from the column, number outside brackets—total number).

Before Fatigue Test									
	Jump 1 *n* (%)			Jump 2 *n* (%)			Jump 3 *n* (%)		
	Study Group I	Study Group II	Control Group	Study Group I	Study Group II	Control Group	Study Group I	Study Group II	Control Group
Correct landing	0 _a_	5 _a_ (38.5)	23 _b_ (79.3)	2 (50.0)	9 (69.2)	24 (82.8)	3 (75.0)	8 (61.5)	20 (69.0)
Slight shift	1 _a_ (25.0)	1 _a_ (7.7)	5 _a_ (17.2)	1 (25.0)	2 (15.4)	3 (10.3)	0	2 (15.4)	6 (20.7)
Significant shift	2 _a_ (50.0)	1 _a,b_ (7.7)	0 _b_	0	1 (7.7)	1 (3.4)	1 (25.0)	2 (15.4)	2 (6.9)
Unstable	1 _a,b_ (25.0)	6 _b_ (46.2)	1 _a_ (3.4)	1 (25.0)	1 (7.7)	1 (3.4)	0	1 (7.7)	1 (3.4)
Test result	*p* < 0.001; *V* = 0.56			*p* = 0.376; *V* = 0.22			*p* = 0.739; *V* = 0.18		

In a Fisher’s exact test context, ‘a’ and ‘b’ are the counts of observations in the cells of a 2 × 2 contingency table, specifically representing the number of instances for a given category

**Table 4:** Frequency analysis with Fisher’s exact test for qualitative jump analysis before fatigue test—lower left extremity.

Before Fatigue Test									
	Jump 1 *n* (%)			Jump 2 *n* (%)			Jump 3 *n* (%)		
	Study Group I	Study Group II	Control Group	Study Group I	Study Group II	Control Group	Study Group I	Study Group II	Control Group
Correct landing	4 (66.7)	3 (25.0)	13 (44.8)	4 (66.7)	7 (53.8)	18 (62.1)	4 (66.7)	8 (61.5)	22 (78.6)
Slight shift	1 (16.7)	4 (33.3)	12 (41.4)	0	3 (23.1)	8 (27.6)	0	2 (15.4)	4 (14.3)
Significant shift	0	3 (25.0)	3 (10.3)	0	2 (15.4)	1 (3.4)	0	3 (23.1)	2 (7.1)
Unstable	1 (16.7)	2 (16.7)	1 (3.4)	2 (33.3)	1 (7.7)	2 (6.9)	2 (33.3)	0	0
Test result	*p* = 0.251; *V* = 0.28			*p* = 0.299; *V* = 0.28			*p* = 0.057; *V* = 0.44		

**Table 5:** Frequency analysis with Fisher’s exact test for qualitative jump analysis before fatigue test—lower right extremity.

After Fatigue Test									
	Jump 1 *n* (%)			Jump 2 *n* (%)			Jump 3 *n* (%)		
	Study Group I	Study Group II	Control Group	Study Group I	Study Group II	Control Group	Study Group I	Study Group II	Control Group
Correct landing	3 (75.0)	5 (41.7)	16 (55.2)	2 (50.0)	7 (53.8)	19 (65.5)	4 (100.0)	12 (92.3)	20 (78.3)
Slight shift	0	1 (8.3)	7 (24.1)	1 (25.0)	1 (7.7)	7 (24.1)	0	1 (7.7)	4 (13.8)
Significant shift	0	3 (25.0)	1 (3.4)	1 (25.0)	2 (15.4)	3 (10.3)	0	0	3 (10.3)
Unstable	1 (25.0)	3 (25.0)	5 (17.2)	0	3 (23.1)	0	0	0	2 (6.9)
Test result	*p* = 0.311; *V* = 0.29			*p* = 0.113; *V* = 0.33			*p* = 0.800; *V* = 0.22		

**Table 6:** Frequency analysis with Fisher’s exact test for qualitative jump analysis after fatigue test—lower left extremity.

After Fatigue Test									
	Jump 1 *n* (%)			Jump 2 *n* (%)			Jump 3 *n* (%)		
	Study Group I	Study Group II	Control Group	Study Group I	Study Group II	Control Group	Study Group I	Study Group II	Control Group
Correct landing	3 (50.0)	6 (50.0)	17 (58.6)	3 (50.0)	7 (53.8)	17 (63.0)	5 (83.3)	9 (69.2)	17 (63.0)
Slight shift	1 (16.7)	2 (16.7)	7 (24.1)	2 (33.3)	2 (15.4)	3 (11.1)	1 (16.7)	0	5 (18.5)
Significant shift	0	2 (16.7)	3 (10.3)	0	3 (23.1)	7 (25.9)	0	2 (15.4)	3 (11.1)
Unstable	2 (33.3)	2 (16.7)	2 (6.9)	1 (16.7)	1 (7.7)	0	0	2 (15.4)	2 (7.4)
Test result	*p* = 0.656; *V* = 0.22			*p* = 0.250; *V* = 0.28			*p* = 0.604; *V* = 0.23		

**Table 7:** Descriptive statistics for a mean of three jumps for lower right extremity (M—mean, SD—standard deviation, SE—standard error, LL—lower level of confidence interval, UL—upper level of confidence interval).

					95% CI	
		M	SD	SE	LL	UL
TTS—before fatigue test	Study group I	1.34	0.18	0.17	1.01	1.67
	Study group II	1.65	0.28	0.10	1.46	1.84
	Control group	1.42	0.36	0.06	1.30	1.54
TTS—after fatigue test	Study group I	1.46	0.57	0.25	0.96	1.96
	Study group II	1.50	0.49	0.14	1.21	1.79
	Control group	1.52	0.49	0.09	1.34	1.71

**Table 8:** Descriptive statistics for a mean of three jumps for lower left leg.

					95% CI	
		M	SD	SE	LL	UL
TTS [s] before fatigue test	Study group I	1.31	0.43	0.18	0.96	1.66
	Study group II	1.62	0.35	0.12	1.37	1.87
	Control group	1.47	0.45	0.08	1.32	1.63
TTS [s] after fatigue test	Study group I	1.36	0.27	0.19	0.98	1.74
	Study group II	1.50	0.60	0.14	1.23	1.77
	Control group	1.45	0.44	0.09	1.27	1.62

## Data Availability

The data presented in this study are available on request from the corresponding author due to lack of possibility to store them in repository.
